# Adjusting for HLA-DRβ1 in a genome-wide association analysis of rheumatoid arthritis and related biomarkers

**DOI:** 10.1186/1753-6561-3-s7-s12

**Published:** 2009-12-15

**Authors:** Abigail G Matthews, Jia Li, Chunsheng He, Jurg Ott, Mariza de Andrade

**Affiliations:** 1 Laboratory of Statistical Genetics, Rockefeller University, Box 192, 1230 York Avenue, New York, NY 10065, USA; 2 Department of Health Sciences Research, Mayo Clinic, 200 First Street Southwest, Rochester, MN 55905, USA; 3 Beijing Institute of Genomics, Chinese Academy of Sciences, Number 7 Bei Tu Cheng West Road, Chaoyang District, Beijing 100029, PR China

## Abstract

**Background:**

There is a long-established association between rheumatoid arthritis and HLA-DRβ1. The shared epitope (SE) allele is an indicator of the presence of any of the HLA-DRβ1 alleles associated with RA. Other autoantibodies are also associated with RA, specifically rheumatoid factor IgM (RFUW) and anti-cyclic citrullinated peptide (anti-CCP).

**Methods:**

Using the Genetic Analysis Workshop 16 North American Rheumatoid Arthritis Consortium genome-wide association data, we sought to find non-HLA-DRβ1 genetic associations by stratifying across SE status, and using the continuous biomarker phenotypes of RFUW and anti-CCP. To evaluate the binary RA phenotype, we applied the recently developed FP test and compared it to logistic regression or a genotype count-based test. We adjusted for multiple testing using the Bonferroni correction, the Q value approach, or permutation-based *p*-values. A case-only analysis of the biomarkers RFUW and anti-CCP used linear regression and ANOVAs.

**Results:**

The initial genome-wide association analysis using all cases and controls provides substantial evidence of an association on chromosomes 9 and 2 within the immune system-related gene *UBXD2*. In SE-positive subjects, many single-nucleotide polymorphisms were significant, including some on chromosome 6. Due to very few SE negative cases, we had limited power to detect associations in SE negative subjects. We were also unable to find genetic associations with either RFUW or anti-CCP.

**Conclusion:**

Our analyses have confirmed previous findings for genes *PTPN22 *and *C5*. We also identified a novel candidate gene on chromosome 2, *UBXD2*. Results suggest FP test may be more powerful than the genotype count-based test.

## Background

Genetic associations between rheumatoid arthritis (RA) and HLA loci have been known for some 30 years [1] but only in recent times, with the availability of dense genetic maps, have associations with other genomic areas been discovered. An affected-sibpair analysis demonstrated significant linkage of RA with single-nucleotide polymorphisms (SNPs) at chromosomes 2*q*33 and 11*p*12 with additional, nonsignificant, linkage to SNPs on chromosomes 4, 7, 12, 16, and 18 [2]. Genome-wide association studies have confirmed and refined associations with HLA variants [3-5], specifically with HLA-DRβ1. The high risk alleles for HLA-DRβ1, commonly referred to as the shared epitope (SE), were found to be highly associated with RA [6,7]. Thus, individuals can be characterized as SE positive (at least one high risk allele) or SE negative (no high risk alleles). Lee et al. [8] have recently shown that there are associations within the major histocompatibility complex (MHC) that are independent of HLA-DRβ1. Autoantibodies, such as rheumatoid factor IgM (RFUW) and anti-cyclic citrullinated peptide (anti-CCP) have been shown to co-occur with RA [9-11]. Thus, genetic predictors of these two biomarkers may also be of interest.

Using unrelated cases and controls from the North American Rheumatoid Arthritis Consortium (NARAC) [12] provided by the Genetic Analysis Workshops 16 and previously analyzed by Plenge et al. [4], we sought to find non-HLA-DRβ1 genetic associations separately in SE positive and SE negative individuals. The literature does not contain any analysis of genome-wide association adjusted for SE status; thus, our primary objective was to stratify the genetic analyses by SE status. Our aims are to identify genomic regions in SE negative individuals that may act independently of the SE in RA development, to evaluate any potential effect modification or epistasis with HLA-DRβ1 in SE positive individuals, and to identify new genomic regions associated with the continuous phenotypes of RFUW and anti-CCP. We also sought to implement the recently proposed FP test [13] and compare it to other methods of analysis.

## Methods

### Quality control

As previously noted, heterogeneity in this dataset is largely due to SNPs with incomplete genotyping [4]. Thus, we focused on the subset of SNPs with call rates exceeding 95.5%. Markers with only one genotype observed were excluded as well as those with a minor allele frequency (MAF) less than 5%. Further, SNPs with the χ^2 ^for Hardy-Weinberg disequilibrium greater than 50 were also removed from consideration. Individuals with more than 20% of SNPs missing were excluded. Only autosomal SNPs were analyzed in this study.

### Statistical analysis

As an initial pass through the data, a genome-wide association analysis was performed using the FP test and a genotype-based χ^2 ^test of association for all non-chromosome 6 SNPs. These SNPs were ignored at first because of the potential for disequilibrium with the HLA-DRβ1. These analyses considered all cases and controls, that is, the number of SE alleles was not accounted for in any way. The FP test evaluates differences in allele frequencies *and *inbreeding coefficients between cases and controls. Both statistics were implemented using the *sumstat *program [14] which computes permutation-based *p*-values such that further adjustment for multiple testing is unnecessary. For all analyses, 5000 permutations were used.

For the genome-wide association analyses of RA adjusting for SE status, we performed multiple methods of analyses separately in those who are SE positive and those who are SE negative. Three different analytic methods were applied to each SNP. First, logistic regressions were implemented in PLINK [15] with sex as an additional covariate. For these results, multiple comparisons were adjusted for using the Bonferroni correction as well as the Q value approach [16], which controls the false discovery rate (FDR). Due to the large number of statistically significant SNPs in this analysis, all subsequent analyses do not consider any SNPs on chromosome 6. As in the initial analyses, both the χ^2 ^test of genotype-based association and the FP test were implemented in *sumstat *using 5000 permutations.

Genetic analysis of the two biomarkers RFUW and anti-CCP were conducted in a similar fashion, but only considering the 868 cases. Since these outcome variables are continuous, linear regression with sex as an additional covariate was implemented using PLINK. The quantile rank transformation was applied to both outcomes to satisfy distribution assumptions. Multiple testing was adjusted for using the Bonferroni and Q value approaches. ANOVAs were used to analyze the biomarkers separately and jointly using *sumstat *and its permutation-based *p*-values. Due to the normality assumptions of this approach, anti-CCP and RFUW levels were transformed using the normal quantile transformation. For each SNP, three tests were considered: i) ANOVA considering only anti-CCP, ii) ANOVA considering RFUW alone, and iii) the maximum ANOVA statistic for anti-CCP and RFUW. The permutations implemented by *sumstat *allow for control of the type I error rate for the maximum statistic and multiple testing simultaneously.

## Results

### Quality control

Initially, 531,689 autosomal SNPs were available for analysis. Due to only one genotype being observed, 1,771 SNPs were removed. With regards to substantial deviations from Hardy-Weinberg equilibrium, 22 SNPs were also removed. Further, another 38,829 SNPs did not pass quality control because their MAF was less than 5%. After also removing SNPs with call rates less than 95.5%, we were able to consider 481,486 autosomal SNPs. For the genotype-based analyses and FP tests, chromosome 6 SNPs were excluded, leaving 338,671 for this set of analyses. Because the lowest call rate was over 97%, we considered all 2062 subjects.

### Initial genome-wide association analysis

Twelve non-chromosome 6 SNPs were statistically significant in the initial pass through the data using the FP or χ^2 ^test of association with genotype counts. Table 1 describes these "best" non-chromosome 6 SNPs and the permutation-based *p*-values for both tests. There are six SNPs on chromosome 9 that are statistically significant at the 0.05 level, all within 65 kb of each other. The remaining significant SNPs are located on chromosomes 1, 2, 8, and 10.

**Table 1:** Analysis of non-chromosome 6 SNPs using all subjects

SNP ID	Chromosome	Location (bp)	FP (*p*-value)	Genotype-based χ^2 ^(*p*-value)
rs2476601	1	114089610	<0.0001	<0.0001
rs2900180^a^	9	120785936	0.003	0.014
rs3761847	9	120769793	0.006	0.031
rs881375	9	120732452	0.011	0.046
rs2671692	10	49767825	0.011	0.057
rs1953126	9	120720054	0.012	0.053
rs10760130	9	120781544	0.013	0.064
rs4921720	8	20397678	0.024	0.123
rs11204117	8	20396191	0.026	0.131
rs10106243	8	20388180	0.031	0.153
rs1446585	2	136241211	0.034	0.163
rs10985073	9	120723409	0.035	0.165

^a^Also significant using FP test only on individuals with shared epitopes.

### Adjusting for SE status

**Figure 1:**
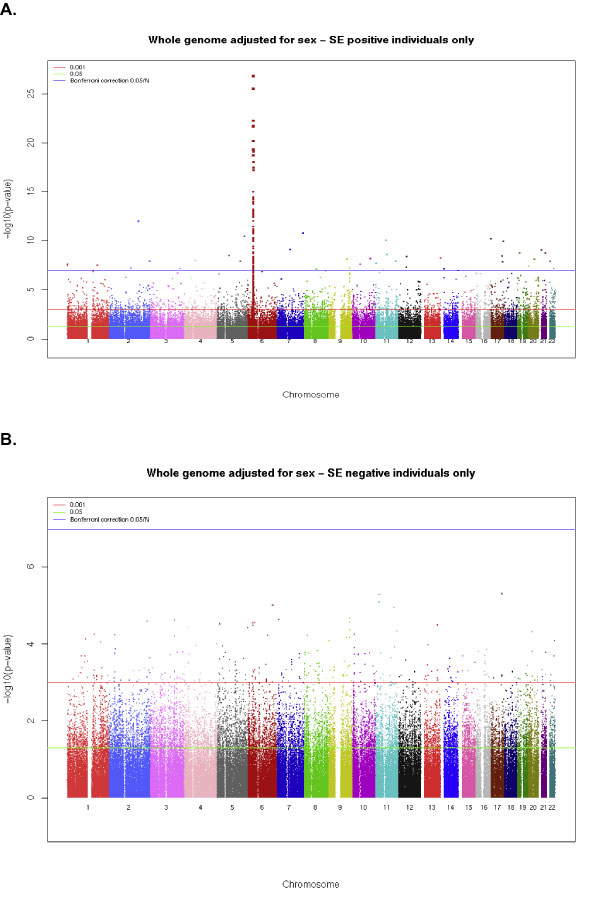
**Manhattan plots from the logistic regression analysis of RA stratifying by SE status**. A, Results from the regression analyses in SE positive subjects adjusted for sex using all autosomal SNPs, including chromosome 6. B, Results from the regression analyses in SE negative subjects adjusted for sex using all autosomal SNPs, including chromosome 6.

Of the 868 cases, 810 were SE positive; and of the 1194 controls, only 541 were SE positive. Figure 1A gives the results of the logistic regression analyses of all autosomal SNPs in the 1351 individuals who are SE positive. Using the Bonferroni correction, there were statistically significant SNPs on virtually every autosome. The most highly significant SNPs are located on chromosome 6 in the HLA region, with several *p*-values less than 10^-25^. Figure 1B plots the results of the logistic regression analyses of all autosomal SNPs in the SE negative participants. None of the over 480,000 SNPs were statistically significant in the SE negative individuals after adjustment for multiple comparisons with the Bonferroni or Q value corrections. When the chromosome 6 SNPs are excluded from analysis and the FP test is implemented, only one SNP was statistically significant for SE positive subjects: rs2900180 (*p *= 0.0196) on chromosome 9. In SE negative individuals, no SNPs were statistically significant (all *p *> 0.35) using the FP test.

### Analysis of anti-CCP and rheumatoid factor IgM

The quantitative trait analyses of anti-CCP and RFUW were performed using cases only because this information was only available on the 868 subjects with RA. Not a single SNP was significant using any of the methods above. Due to the small sample size and the fact that all subjects had RA, we may have lacked power to detect any genetic association with levels of anti-CCP or RFUW.

## Discussion

When considering all cases and controls regardless of SE status, 12 SNPs were statistically significant and spread across chromosomes 1, 2, 8, 9, and 10. The most significant SNP, rs2476601, is located on chromosome 1 less than 1 Mb from the PTPN22 gene, which has been shown to be associated with RA [14,17]. Six SNPs on chromosome 9 were also significant with the FP test, and they are all located in the same region: 120.72 to 120.79 Mb. These results may be due to high linkage disequilibrium (LD); in fact, three pairs of these SNPs have *r*^2 ^greater than 0.8. This region of chromosome 9, located near the immunity-related complement compound 5 (*C5*) gene, has been previously identified as associated with RA [4,18]. Three new associations are suggested by our analyses. On chromosome 8, three SNPs were statistically significant and are all located within 10 kb of each other. Yet another SNP was significant on chromosome 10 using the FP test, but was not significant using the genotype-based χ^2 ^test. Interestingly, we found evidence of an association on chromosome 2 at rs1446585, which is 35 Mb from a signal reported by Plenge et al. [4]. Our SNP is located within the *UBXD2 *gene, which has been shown to be differentially expressed in the spleen and plays a role in the immune system [19]. This is particularly of interest because RA is an autoimmune disease.

One goal of our genome-wide association analysis was to stratify by SE status, which has not been done previously for a genome-wide association study. Using the FP test on SE positive subjects, only one SNP was statistically significant, namely, rs2900180, which is located within approximately 2 Mb of *C5*. More SNPs were significant in this region near *C5 *when all subjects were considered, but this difference does not imply epistasis between *C5 *and HLA-DRβ1. It may only be due to the decrease in sample size. When restricting our analysis to SE negative individuals, none of the three tests yielded evidence of another gene associated with the development of RA. The increase in risk associated with the shared epitope allele is so great that very few of the cases were SE negative. With only 58 cases available for these analyses, it is not surprising no SNPs were significant. Although one of our main objectives was to identify genomic regions that may act independently on development and progression of RA, we were unable to do so with adequate power.

Our analyses of the biomarkers anti-CCP and RFUW yielded no significant results. Information on these continuous phenotypes was only available for the 868 cases, so the controls were not considered at all. Since all subjects had RA, it may be that there is no genetic association with either biomarker once disease has developed or progressed. RFUW is a measure of disease activity, so the lack of observed association may be due to the study design such that cases are fairly homogeneous in terms of disease severity.

Our final goal was to compare the implementation of the FP test with the other methods employed here. The significance level approach based on the FP test found far fewer SNPs to be statistically significant than the FDR approach (i.e., Q value) based on logistic regression analysis. This may show superiority of the concept of controlling the FDR over the significance level approach, and/or this difference may be due to inclusion in the logistic regression analysis of predictor variables, which were not considered in the FP analyses. Because permutation-based *p*-values were used for the FP and genotype-based χ^2 ^test in Table 1, the fact that the FP has smaller *p*-values than the χ^2 ^test for each SNP indicates the former may be more powerful.

## Conclusion

This study demonstrates that there may be several genes that co-function with HLA-DRβ1 in the development and severity of RA, most likely on chromosome 9, such as *C5*, and other genes within the MHC region on chromosome 6. We have also identified another region on chromosome 2 near the *UBXD2 *gene as a candidate for follow-up study. In addition, we have shown that in order to identify new genomic regions that cause RA in SE negative individuals, an alternative study design must be implemented.

## List of abbreviations used

Anti-CCP: Anti-cyclic citrullinated peptide; FDR: False discovery rate; LD: Linkage disequilibrium; MAF: Minor allele frequency; MHC: Major histocompatibility complex; NARAC: North American Rheumatoid Arthritis Consortium; RA: Rheumatoid arthritis; RFUW: Rheumatoid factor IgM; SE: Shared epitope; SNP: Single-nucleotide polymorphism

## Competing interests

There are no competing interests for AGM, JL, CH, JO or MdA.

## Authors' contributions

For the first set of analyses using all SNPs, MdA specified the methodology and JL implemented the procedures. JO implemented the other analyses designed by himself, AGM and CH. This manuscript was principally written by AGM.
